# Ion Frequency Landscape in Growing Plants

**DOI:** 10.1371/journal.pone.0138839

**Published:** 2015-10-07

**Authors:** Mariusz Pietruszka, Aleksandra Haduch-Sendecka

**Affiliations:** Department of Plant Physiology, Faculty of Biology and Environment Protection, University of Silesia, Katowice, Poland; Hainan University, CHINA

## Abstract

It has been interesting that nearly all of the ion activities that have been analysed thus far have exhibited oscillations that are tightly coupled to growth. Here, we present discrete Fourier transform (DFT) spectra with a finite sampling of tip-growing cells and organs that were obtained from voltage measurements of the elongating coleoptiles of maize *in situ*. The electromotive force (EMF) oscillations (~ 0.1 μV) were measured in a simple but highly sensitive resistor–inductor circuit (RL circuit), in which the solenoid was initially placed at the tip of the specimen and then was moved thus changing its position in relation to growth (EMF can be measured first at the tip, then at the sub-apical part and finally at the shank). The influx- and efflux-induced oscillations of Ca^2+^, along with H^+^, K^+^ and Cl^-^ were densely sampled (preserving the Nyquist theorem in order to ‘grasp the structure’ of the pulse), the logarithmic amplitude of pulse spectrum was calculated, and the detected frequencies, which displayed a periodic sequence of pulses, were compared with the literature data. A band of life vital individual pulses was obtained in a single run of the experiment, which not only allowed the fundamental frequencies (and intensities of the processes) to be determined but also permitted the phase relations of the various transport processes in the plasma membrane and tonoplast to be established. A discrete (quantised) frequency spectrum was achieved for a growing plant for the first time, while all of the metabolic and enzymatic functions of the life cell cycle were preserved using this totally non-invasive treatment.

## Introduction

The physico-chemical nature of wall loosening in growing plants is still an issue that is being investigated [1]. Oscillations appear to be common processes in biological systems in the context of growth [2–3] and may be closely related to the emergence of the phenomenon of life itself. Biological oscillation is a basic mechanism that underlies many biological processes, ranging from cellular signalling to tip growth [4]. The role of ion gradients and fluxes in the study of plant cell growth and elongation in plants, especially Ca^2+^ and protons in the case of pollen tubes, has attracted a great deal of attention [5, for review]. Ca^2+^ is one of most abundant elements in the earth’s crust and plays a wide variety of roles within cells [6]–Ca^2+^ ion concentrations can lead to localised or even plant-wide oscillations that can regulate downstream events. Cells maintain a steady state Ca^2+^ concentration about hundred times greater than its concentration in the extracellular space. Such a large difference in concentration allows for rapid oscillations through the opening of Ca^2+^ channels in the separating membranes. For our purposes, it is important to note that “gating” permits the maintenance of large differences in concentration with the exterior of the cell, as well as in the orchestrated release of Ca^2+^ during signalling events. H^+^-ATPases generate a voltage of about –150 mV across the plasma membranes of plants, which results in an electrochemical driving force of about 300 mV for Ca^2+^. A schematic diagram of the different mechanisms for the passive and active transport of Ca^2+^ is presented in the review article by Martins et al. [6]. The oscillations of Ca^2+^ concentration open and close DMI1 channel, thus leading to a periodic K^+^ ions efflux (ibid). On the other hand, Shabala et al. [7] reported measurements of Ca^2+^ and H^+^ fluxes around the elongation region of corn roots at various pH levels. They noted that oscillations in ion fluxes were always detectable if the length of the observation was long enough. They also concluded that the different oscillatory components of ion fluxes around the roots of a plant appeared to be related to different ion transport systems. Investigations conducted by Zonia et al. [8] indicated that the dynamics of the Cl^-^ ion is an important ingredient in the network of events that regulate pollen tube homeostasis and growth. According to Zonia, oscillatory Cl^-^ efflux at the pollen tube apex plays a role in growth and cell volume regulation. By using an ion-specific vibrating probe the oscillatory growth of pollen tubes has been correlated with oscillatory influxes of the cations Ca^2+^, H^+^ and K^+^.

A key role for understanding the basic (both oscillatory and monotonic) growth mechanisms is attributed to ionic transport across membrane barrier. As was mentioned above, there are countless articles on passive and active transport in plants and the influx and efflux of substances, such as sucrose or hexose or different anions and cations, have been described in detail. Many trials have been undertaken to systematise this knowledge not only on the level of plant kingdom or species, but also for organs, tissues, cells or organelles. One of the most famous is the "Overview of the various transport processes on the plasma membrane and tonoplast of the plant cells", which is presented in Taiz and Zeiger [9] although even here there is no information about the time sequence of the events (processes) in the cell cycle. However, it is highly credible that the time sequence may be fundamental for finding the solution to many discussions among biologists about the most basic mechanisms, such as the role of turgor pressure in the building of the primary wall. In this context, our proposal of a new universal and inexpensive method, which is able to detect and discriminate the ionic frequency landscape of a growing plant, seems to be a milestone in growth physiology. It is clear that the presented method, although not completely autonomous, is drastically limited by the available literature on ionic transport in plants, and the fact that most research is conducted on model plants.

By mentioning only few reports from the abundant literature on ion transport being tightly coupled to growth, we have exemplified that oscillations (periodic motion) are ubiquitous in the elongation zones of growing plant organs. The latter statement is correct regardless of whether we are dealing with the multi-cell elongation zone in maize coleoptiles or roots, or the single-cell (subapical) elongating part of a pollen tube. Based on this observation, we propose a new experimental method, which allowed us to retrieve the basic phase relations and ion oscillation frequencies of an intact *Zea mays* L. plant in a single run of the experiment. This work presents very subtle electrical measurements of growing maize coleoptiles that were obtained using an external (non-invasive) EMF measuring system. The data were compared with other oscillatory electrical and ionic data from other systems and were subjected to frequency spectrum analysis. The results that were obtained–ion frequencies, amplitudes (magnitudes) and phases–were compared with the available literature data for identification. The results are essentially correlative and provide information about the timing of the underlying processes.

## Materials and Methods

### Investigation of Ca^2+^, H^+^, K^+^ and Cl^-^ fluxes in single maize coleoptile in situ

Maize cells were used as the experimental system with which to study ion transport processes and establish the characteristic timing in the elongating cell complex of an individual higher plant using a solenoid-based circuit. Measurements were carried out on multi-cell organs (three-day-old coleoptiles) of maize (*Zea mays* L.) *in situ*. Seeds of maize were grown on a Hoagland’s medium [10] and cultivated in darkness at 27°C. Then they were placed (*in vivo* studies) in artificial pond water (APW) in a dark chamber (S1 Fig) at a fairly constant temperature. The temperature was measured before and after the experiment (24–25°C in which the optimum growth of maize takes place). The pre-incubation time was 30 min, while the duration of the experiment was from one to twenty hours (a four-hour experiment, which was chosen as the most efficient one after many trials, is presented here). The thermally isolated measuring chamber was shielded from the electromagnetic fields of environment. It was covered with aluminium thin foil to create a Faraday cage. Both the chamber and the 6 ½ Digit Precision Multimeter were grounded. The initial length of the seedling and the length increment was read off. In order to optimise the measurement conditions, we performed 39 experiments of different durations. In almost all of the experiments the coil was placed about 3–5 mm below the tip at the beginning of the experiment (S2 Fig). The fragment of maize coleoptiles that was investigated elongated most intensely and was free from cell divisions. Ice was added into an additional container (with an immersed NiCr-NiAl thermocouple) just before the measurement, so that it wouldn’t melt in the course of experiment (S3 Fig). The measurement was usually done after several minutes of the primary stabilisation of the whole system. The growing seedlings remained untouched during this non-invasive experiment and were able to continue to grow and develop leaves after the experiment (S2 Fig).

Both, the co-moving (with the advancing tip) and the fixed reference system (located at the shank) were used. In the case of co-moving frame, the increasing distance of the coil form the tip allowed unique continuous measurements to be performed at the three apical and subapical zones (tip, subapex–intermediate zone, shank) of the same growing sample. In our method, we also omitted the technically demanding problem related to the difficulty of impaling the apex in order to perform the measurements, while still maintaining elongation.

### DFT analysis

The (negative feedback) RL circuit was formed tightly in the form of a solenoid on the growing coleoptile (S2 Fig). The cooper wires in the form of a shielded twisted pair were used to connect to the rest of the system. The net voltage (thermocouple ‘constant’ voltage V plus Lenz-rule–induced V_EMF_ stating that the induced EMF and induced current were in such a direction as to oppose the cause that produced them) was measured (S3 and S4 Figs). The corresponding constitutive relation was used: ${\text{V}}_{\text{EMF}}=\frac{\text{d}\Phi }{\text{dt}}=\frac{\text{d}\left(\text{LJ}\right)}{\text{dt}}=\text{L}\frac{\text{dJ}}{\text{dt}}$. The inductance L = 23.68 μH was calculated from the following data: number of turns n = 118, length 0.75 ± 0.01 cm, radius 0.2 ± 0.01 cm and permeability of a vacuum μ = 4π ∙ 10^−7^ H/m. The coil Cu wire diameter equalled 0.12 mm, Cu connectors 0.16 mm; Cu plates that served as conductance electrons’ reservoir [11–12], with an average volume of 158.36 ± 0.1 mm^3^. The absorption spectrum measurement was possible not only because of the long duration of the experiment (3600 s up to 72000 s) with dense (1 s) sampling, but presumably it was also amplified due to the type of “collective excitation” of many (~ 0.5 ∙ 104, [13]) cells during one cycle. Note, that the synchronicity of different coupled oscillators may, by preventing destructive interference, increase the robustness of the signal [14]. By assuming typical values for dJ ~ 1 pA and dt ~ 1 ms, we got V_EMF_ ~ 10^−13^ V. However, by taking into account, by straightforward calculation, the number of cells in the investigated sample, and assuming about 100 channels per cell, we finally got the estimate V_EMF_ = 10^−6^ V, which is within the reach of our measuring apparatus (μV). To be on the safe side, the measurements were performed in the “ac” and “dc” modes of the voltmeter and were recorded by a computer program (see S5 Fig for the time series). The ‘void’ measurements with an empty coil (with no coleoptile placed inside) were performed twice for reference purposes and the Fourier decomposition revealed no signal.

The absorption lines were then detected *via* the discrete Fourier transform (DFT), see Harris [15] and Dieckmann [16], and the read-off frequencies were associated with and compared to the literature data (S1–S4 Tables, S6 and S7 Figs). Recognition of these extremely small signals was possible because the duration of the experiment was long enough compared to the basic period of the investigated signal, and dense sampling. For example, a period *T* of about 16.5 seconds (maximum of Ca^2+^ influx) was encountered 873 times in a four-hour probe. If any regularly occurring event happened every 16.5 seconds within the measured time series, it would clearly be detected by the discrete Fourier transform to produce a spectral line that was connected with this event.

### System stabilisation and cross-correlations

System stabilisation, which was controlled by cross–correlations, is shown in S8 Fig. The system that was investigated stabilised after the first hour of the measurement (Figs B and C in S8 Fig). Closer examination revealed that the corresponding two time series produced the auto-correlation triangle instead of the usual cross-correlation, which is presented in Fig D in S8 Fig. This outcome delivers a convincing argument for the reliability of this method and the reproducibility of the results, thus supporting the proposed measurement method.

## Results

Non-invasive resistor–inductor circuit (RL) technique was used to measure H^+^, K^+^, Ca^2+^ and Cl^-^ ion fluxes at the extending coleoptile wall surface during maize growth.

### Determination of ion frequencies and phases

**Fig 1:**
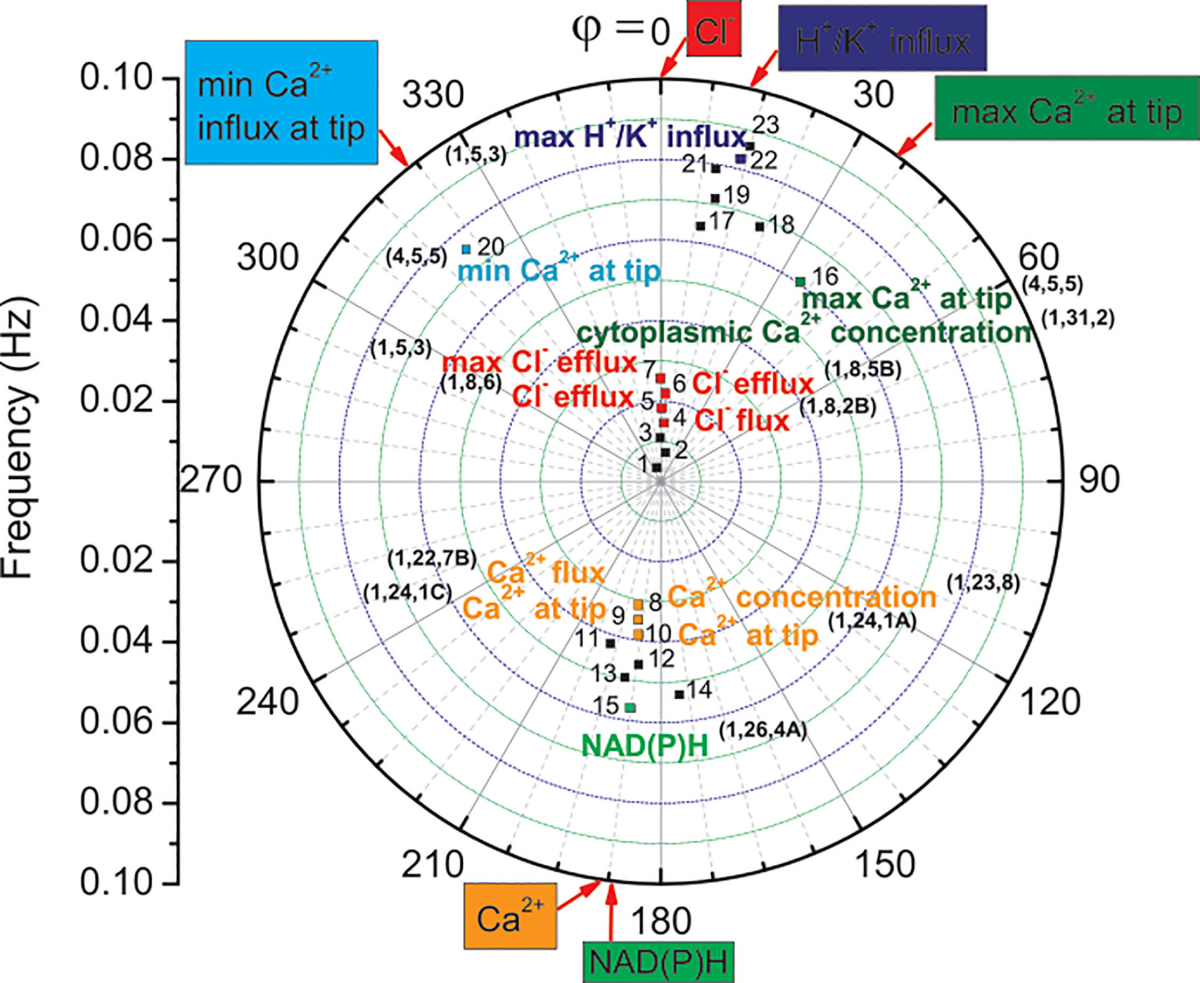
Sequence of events in a maize (*Zea mays* L.) growing tip (subapical part) as indicated in a “radar display” (polar coordinates) chart. The identified frequencies (square dots)–radial coordinate; phase offsets in degrees–angular coordinate (phase φ = 0 was assumed for Cl^-^ efflux). Colour squares–data identified in the literature; black squares–unidentified data, which may be associated with numerous transport processes such as those presented in Fig 6.14 in Taiz and Zeiger [9]. Number coding by the upper indices: (Sn Table, article reference number, figure in the quoted article). Compare with Fig 3 in Holdaway-Clarke and Hepler [5].

Fluxes of H^+^, K^+^, Ca^2+^ and Cl^-^ were measured at the elongating zone of growing maize coleoptile using negative feedback RL circuit (S1–S4 Figs). Discrete Fourier analysis of the time series that were obtained allowed the frequencies and phases of pulsatile ionic fluxes that were active during elongation growth, which are shown in S7 Fig, to be decoded. In order to categorise the type of ions that took part in the growth event, the data was deciphered using S1–S4 Tables. The result that is based on this procedure is presented in Fig 1.

### Determination of amplitudes. Ion oscillation spectrum

**Fig 2:**
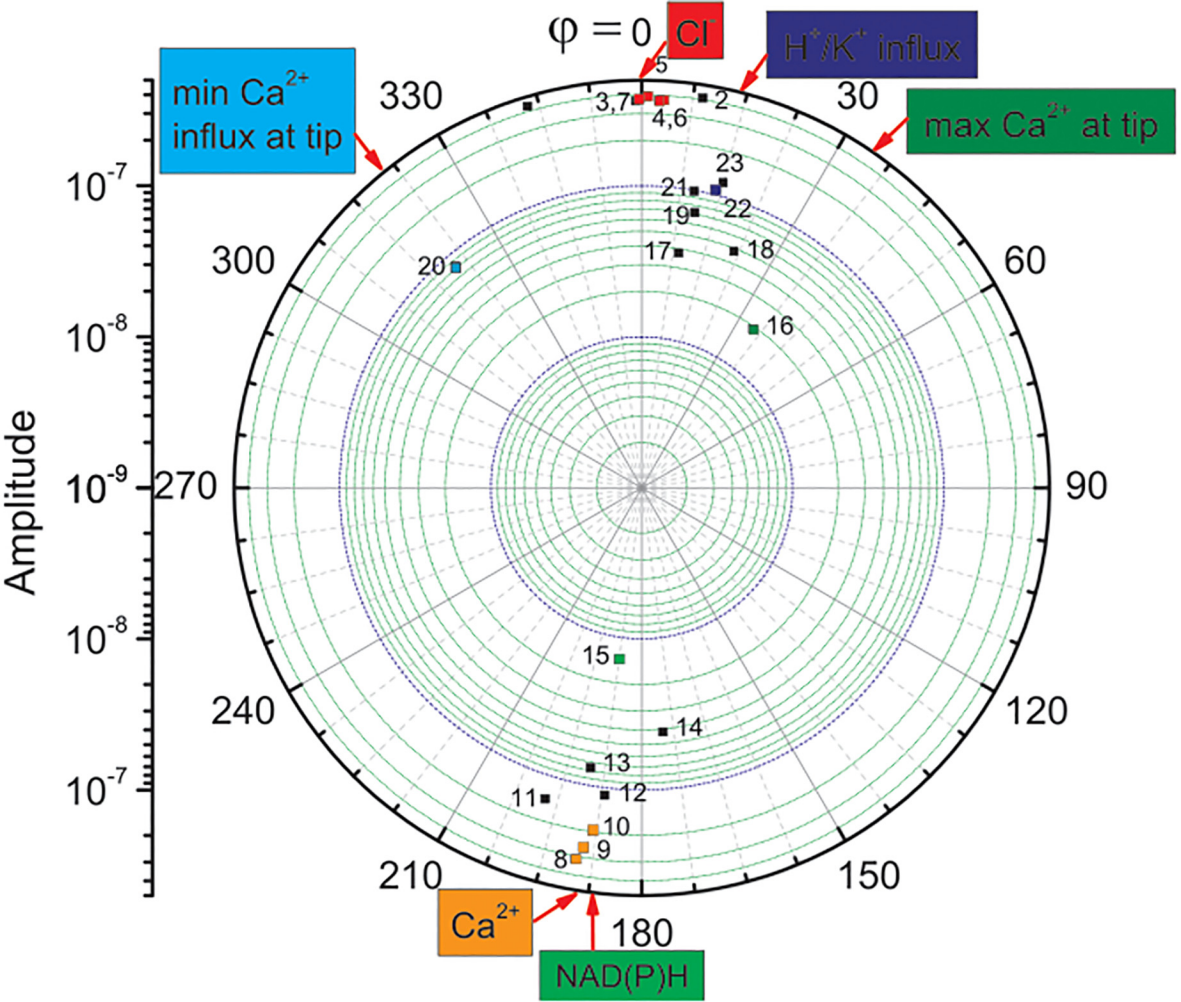
Amplitudes (intensities) of maize (*Zea mays* L.) ionic fluxes in the growing tip presented in polar coordinates. Identified intensities (square dots)–radial coordinate; phase offsets in degrees–angular coordinate (phase φ = 0 is assumed for Cl^-^ efflux). Colour squares–data identified in the literature; black squares–unidentified data, which, however, may be associated with the numerous transport processes that are presented in Fig 6.14 in Taiz and Zeiger [5]. Number coding is the same as in Fig 1.

**Fig 3:**
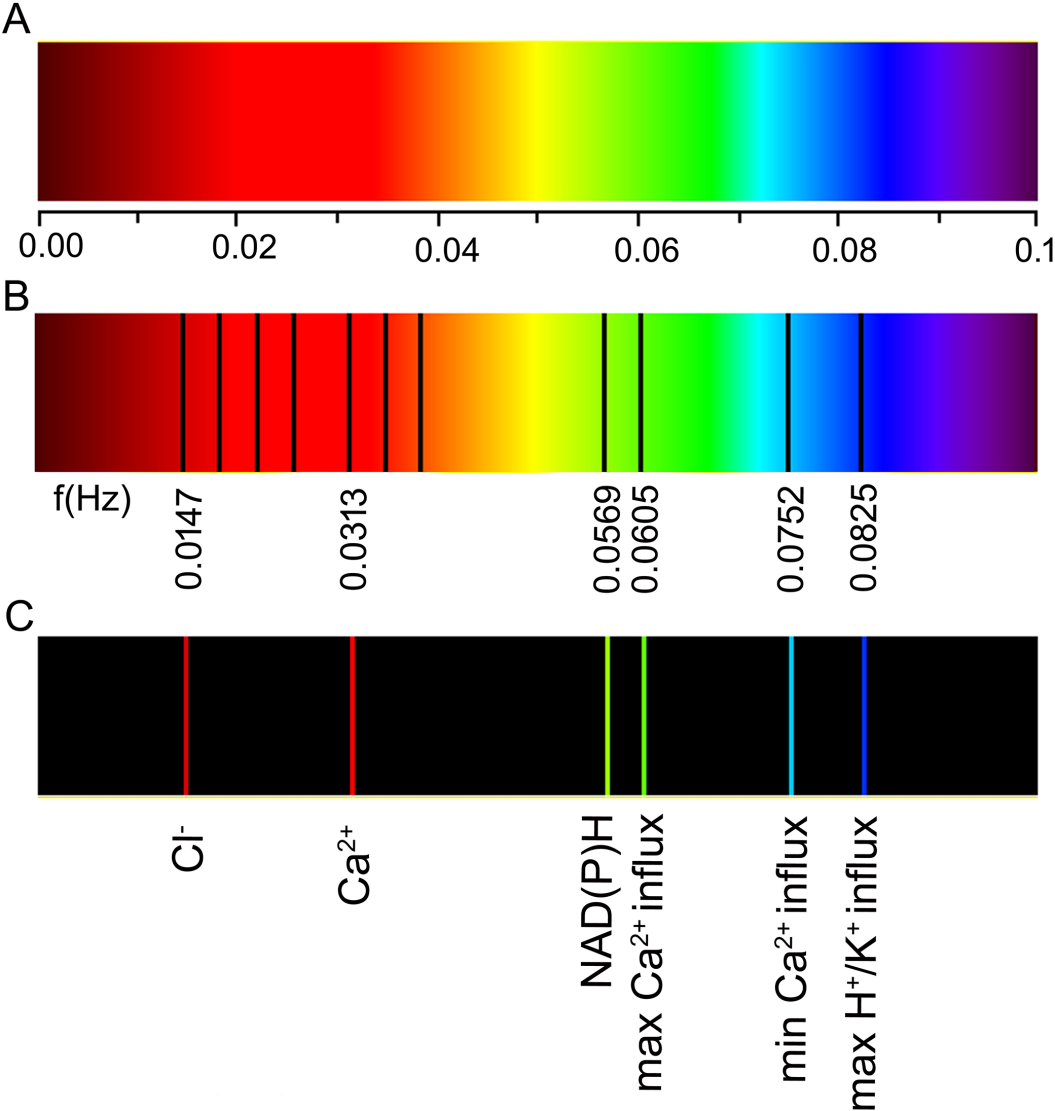
Spectral landscape of a growing maize (*Zea mays* L.) coleoptile. **(A)** Frequency scale in Hz. (**B)** The absorption spectrum is divided into a number of spectral series (basic frequency values are indicated and the higher harmonics that were identified for Cl^-^ and Ca^2+^ are shown). Note the Ca^2+^ base frequency that is slightly blue-shifted. (**C)** Absorption spectrum of basic frequencies for the various ion fluxes that were obtained in our experiment and identified through a comparison with the literature data. Higher harmonics (recognised in the literature and collected in S1–S4 Tables) are shown in B: three lines above 0.0147 Hz for Cl^-^ and two lines above 0.0313 Hz for Ca^2+^.

DFT analysis also delivered the intensities of the processes (magnitudes) that describe the relative strength of the actual process and phase shifts. The amplitudes at given frequencies and the corresponding phases that were obtained *via* DFT analysis are presented in S7 Fig. The data displayed in S7 Fig was assembled in a polar coordinates chart, Fig 2. Some of this data was identified by a comparison with the literature data, which is collected in S1–S4 Tables. The resulting full spectrum of the oscillating ionic fluxes was created in Fig 3 from the corresponding data points in Figs 1 and 2.

## Discussion

Presently, two basic methods that are used to determine the ionic currents and fluxes in plants are reported in the literature. The one is the voltage clamp (patch clamp) method [17], while the other is the ion-specific vibrating probe [18, for review]. The patch clamp technique is a laboratory technique in electrophysiology that allows the study of single or multiple ion channels in cells. The patch clamp technique is a refinement of the voltage clamp and can be applied to a wide variety of cells including plant cells. Electrophysiological techniques that use glass microelectrodes such as voltage clamping can be used on single cells but are non-specific. On the other hand ion-selective probes are glass microelectrodes that contain a small amount of ionophore that is permeable to a particular ion at their tip [18]. The electrode is therefore sensitive to changes in the concentration of this ion. If the probe tip is moved between two points in a concentration gradient of this ion at a low frequency, then the electrochemical potential of the solution inside the electrode fluctuates in proportion to the size of the ion gradient. This fluctuation is amplified and recorded and is used to calculate the actual ion flux using Fick’s law of diffusion [18].

In the non-invasive resistor–inductor circuit (RL) technique, as described in this article, the discrete Fourier transform (DFT) was used for the data analysis. The Fourier spectrum delivers information about the amplitudes, frequencies and phases of the basic cosines that span the function of which an interval that has a length of an integer number of periods is being sampled. Reconstruction of the original shape pulse function (time series) can be completed from a superposition of cosine functions with amplitudes and phases. The Fourier analysis technique is based on the fact that a signal can be decomposed into a sum of cosine or sine functions with periods that are a multiple of the total recording time. A certain periodicity in a recording is reflected by the large weight (Fourier coefficient) that multiplies the cosine or sine function with the respective periodicity in the Fourier decomposition (Fourier series). The value of these Fourier coefficients as a function of their period (or frequency) is called the Fourier transform. Since the Fourier transform is a complex quantity, the power of the Fourier series is usually calculated and is a real quantity [19]. Here, we used the amplitudes, frequencies and phases as the basic ingredients of the spectral analysis [16]. Even though we were able to determine the amplitudes, frequencies and phases of the various ionic fluxes that are present during plant cell growth, the method is not ion-specific and requires auxiliary data that can be obtained by other methods.

The non-invasive, ion-specific, vibrating-electrode probe technique was used, e.g., by Zonia et al. [8] to measure the Cl^-^ ion flux on the extracellular surface during (tobacco and lily) pollen tube growth. The Cl^-^ efflux oscillations at the apex were correlated with the growth rate oscillations that were observed. Similar correlations may possibly emerge in the growing coleoptiles of maize under the condition of the orchestrated action of various ion events thus resulting in growth. Such correlations, however, ought to be sought in experiments that use time-lapse images (CCD camera) that are recording the macroscopical growth of the coleoptile and the subtle changes of the fluorescence- labelled marker genes that are related to tip growth such as the microfilament (e.g. [20]) and the proposed RL-technique simultaneously.

As an aside, we note that the usual problem of the limited temporal resolution of data acquisition, which has hitherto prevented a more detailed analysis, was eliminated in our method by the dense sampling that we applied by using an acquisition interval of 1 s and the sufficiently long duration of the experiment (up to 72000 separate measurements in a single run).

Moreover, it is important to note that the lowest lying frequencies in S9 Fig are very close to the basic frequency f = 0.066 Hz that was obtained independently by theoretical and experimental methods for Ca^2+^ oscillations for a lily in Fig 5, as presented in [21]. Moreover, this basic frequency is almost identical to the re-analysed data in S9 Fig for lily pollen tubes and for that obtained for maize in this work, line *f* = 0.064 Hz in S7 Fig. This may indicate that this lowest lying frequency for Ca^2+^ is the same not only for these two species, but may even be common for other plants.

## Conclusions

Based on the proposed technique, a universal behaviour can be observed in the plant kingdom. Comparison with the literature data (Methods) revealed the existence of equal frequencies of the ionic channel fluxes either for growing single-cell pollen tubes or the multi-cell organs of higher plants. Moreover, phase shift analysis indicates that certain (ionic) processes occur with similar timings.

The new measurement method that is presented, though not entirely autonomous, was focused on finding a complete landscape (spectrum) of well-defined frequencies, intensities and phases of the oscillating ionic fluxes in plants. This turned out to be possible in a single run of a one-penny experiment (copper bar India average price $9.0339/kg gives 0.1 cent/0.1 g solenoid [metalprices.com, 14/04/2015]) for an intact growing plant (*Zea mays* L.). Therefore, this method may be useful for the further analysis of the plant cell cycle, at least as a complementary technique to other well established methods (e.g. patch-clamp, vibration electrode) of the investigation of ionic currents in plant physiology, especially since we are well aware of how difficult it is to identify and characterise any single transport process. Further development of this experimental method may include simultaneous EMF and elongation (volumetric) growth measurements in order to interrelate the underlying microscopic processes with macroscopic observations.

## Supporting Information

S1 Fig Experimental setup. Growing maize (*Zea mays* L.) coleoptile was placed in a thermally isolated (Styrofoam) Faraday cage (Al film) that was situated on a heavy marble plate in order to filter high-frequency ground oscillations.
(TIF)

S2 Fig Maize (*Zea mays* L.) sample measurement *in situ*. During the experiment the solenoid was moved from the tip to a more basal position of a coleoptile. The Cu connectors (wires), which were connected to the experimental setup, are visible. **A.** The investigated sample that was prepared for the experiment–the solenoid is initially placed 5 mm below the tip of a three-day-old seedling. **B.** Photograph taken after a 24 hours measurement. The solenoid was moved to a position of 6.25 mm below the tip. The coleoptile elongation equalled 14.85 mm during the experiment.
(TIF)

S3 Fig Schematic diagram of the measuring apparatus: Cu solenoid (inductance *L* ~ 23 μH), which was wound up tightly on the maize coleoptiles in the apical part, and was connected to the high precision digital multimeter (Tektronix DMM 4040 6 ½ Digit Precision Multimeter) *via* Cu square plates [12] and to the NiCr-NiAl thermocouple that was completely immersed in the ice container, which formed the parallel electric circuit. The voltage was measured and recorded through a RS232/USB interface and the data acquisition was completed on a computer using the Python code (.py).
(PNG)

S4 Fig The solenoid that was used for measurements. The elongating zone of the coleoptile is shown in green. The figure is based on the Shipway and Shipway [22] solenoid properties calculator.
(TIF)

S5 Fig Electromotive force U (V) as a function of time (s). The original data points measured in the dc multimeter mode. The inset shows an oscillatory, step-wise signal structure.
(TIF)

S6 Fig Logarithmic amplitude of a pulse spectrum. The measurement was performed below the coleoptile tip. A sample seed immersed in the APW. Experiment duration 3600 s; sampling 1s, number of solenoid turns n = 118. Compare with Dieckmann [16].
(TIF)

S7 Fig Logarithmic amplitude and phase angle of a pulse spectrum with the frequencies indicated. (TIF)

S8 FigLogarithmic amplitude of a pulse spectrum and corresponding phases.Experiment stabilisation (steady state) is shown in the charts: DFT spectrum was calculated after (A) 1 hour (B) 2 hours (C) 3 hours. (D) Cross-correlations of B and C as a function of time lag (for DFT amplitudes–see main chart, and for the voltage time series–the inset). Cross-correlation is a measure of the similarity of two waveforms as a function of a time-lag that is applied to one of them. By definition, for continuous functions *f* and *g*, the cross-correlation is defined as:
$$(f*g)(\tau )\equiv \int_{-\infty }^{\infty }f^{*}(t)g(t+\tau )dt\;.$$
where f* denotes the complex conjugate of *f* and *τ* is the time lag. In autocorrelation, which is the cross-correlation of a signal with itself, there will always be a peak at a lag of zero. Apparently, the cross-correlation of B and C seem to fulfil this definition (D), even though the experimental data originated from the subsequent (1 hour time delay) measurements. The latter result gives strong support to the experimental method that is introduced in this work.(TIF)

S9 Fig Linear interpolation *T*_n_ = *A + B*n of a basic period and higher harmonics for Ca^2+^ signal for lily. Data based on S3 Table (1). *T*_1_ = 11.67 + 2.22 = 13.89 s (*f*_1_ = 0.072 Hz); *T*_2_ = 11.67 + 2 ∙ 2.22 = 16.11 s (*f*_2_ = 0.062 Hz)–these lowest lying frequencies are close to the basic frequency *f* = 0.066 Hz that was obtained by Pietruszka and Haduch-Sendecka [21] for the Ca^2+^ oscillations that are shown in Fig 5.
(TIF)

S1 Table Ion influx and efflux events and corresponding time periods and frequencies in pollen tubes. *n*–denotes the number of data that were read off from the indicated source. *T*–stands for the oscillation period, while *f* denotes the corresponding frequency.
(XLS)

S2 Table Ion influx and efflux events and corresponding time periods and frequencies in maize. (XLS)

S3 Table Data from S1 and S2 Tables that were used for analysis (*T*–period of oscillations). (XLS)

S4 Table Influx and efflux events and the corresponding phases and frequencies for a lily pollen tube. (XLS)

## References

[1] Breidwood L, Breuer C, Suigimoto K. My body is a cage: mechanisms and modulation of plant cell growth. New Phytol 2014; 201: 388–402. 10.1111/nph.12473 24033322

[2] McKane AJ, Nagy JD, Newman TJ, Stefanini MO. Amplified biochemical oscillations in cellular systems. J Stat Phys 2007; 128: 165–191.

[3] Martin O, Penate L, Alvare A, Cardenas R, Horvath JE. Some possible constraints for life's origin. Orig Life Evol Biospheres 2009; 39: 533–544. 10.1007/s11084-009-9170-9 19554472

[4] Friesen WO, Block GD. What is a biological oscillator? Am J Physiol 1984; 246: R847–53. 6742159 10.1152/ajpregu.1984.246.6.R847

[5] Holdaway-Clarke TL, Hepler PK. Control of pollen tube growth: role of ion gradients and fluxes. New Phytol 2003; 159: 539–563. 10.1046/j.1469-8137.2003.00847.x 33873604

[6] Martins TV, Evans MJ, Woolfenden HC, Morris RJ. Towards the physics of calcium signalling in plants. Plants 2013; 2: 541–588. 27137393 10.3390/plants2040541 PMC4844391

[7] Shabala SN, Newman IA, Morris J. Oscillations in H^+^ and Ca^2+^ ion fluxes around the elongation region of corn roots and effects of external pH. Plant Physiol 1997; 113: 111–118. 12223594 10.1104/pp.113.1.111 PMC158121

[8] Zonia L, Cordeiro S, Tup J, Feijó JA. Oscillatory chloride efflux at the pollen tube apex has a role in growth and cell volume regulation and is targeted by inositol 3, 4, 5, 6-tetrakisphosphate. Plant Cell 2002; 14: 2233–2249. 12215517

[9] Taiz L, Zeiger I. Plant Physiology 4th ed. Sinauer Associates, Inc., Publishers. 2006; Chapter 6: 108–113.

[10] Hoagland DR, Arnon DI. The water-culture method for growing plants without soil. Circular. California Agricultural Experiment Station 1950; 347: 32.

[11] Matlak M, Pietruszka M, Rówiński E. Experimental method to detect phase transitions *via* the chemical potential. Phys Rev B 2001; 63: 052101–1–052101–3.

[12] Matlak M, Pietruszka M. Phase transitions detection by means of a contact electrode. Phys Stat Solidi (b) 2004; 241: 163–169.

[13] Berg JM, Tymoczko JL, Stryer L. Biochemistry. 5th ed. Freeman & Company 2009; Chapter 13: 345–357.

[14] Kroeger JH, Geitmann A. Pollen tube with more viscous cell walls oscillate at lower frequencies. Math Model Nat Phenomena 2013; 8: 25–34.

[15] Harris CM. The Fourier analysis of biological transients. J Neurosci Methods 1998; 83: 15–34. 9765048 10.1016/s0165-0270(98)00080-6

[16] Dieckmann A. Amplitude and phase of a discrete Fourier spectrum ELSA, Physikalisches Institut der Universitat Bonn 2011 http://pi.physik.uni-bonn.de/~dieckman/DFT/DFT.html (Date of access: 15/05/2014).

[17] Sakmann B, Neher E. Patch clamp techniques for studying ionic channels in excitable membranes. Annu Rev Physiol 1984; 46: 455–472. 6143532 10.1146/annurev.ph.46.030184.002323

[18] Reid B, Zhao M. Ion-selective self-referencing probes for measuring specific ion flux. Comm Integrative Biology 2011; 4: 524–527. 10.4161/cib.4.5.16182 PMC3204119 22046453

[19] Zerzour R, Kroeger JH, Geitmann A. Micro-indentation reveals spatially confined dynamic changes in mechanical cell wall properties during plant cell morphogenesis. Develop Biol 2009; 334: 437–446. 10.1016/j.ydbio.2009.07.044 19666018

[20] Chebli Y, Keneda M, Zerzour R, Geitmann A. The cell wall of the Arabidopsis pollen tube–spatial distribution, recycling, and network formation of polysaccharides. Plant Physiol 2012; 160: 1940–1955. 10.1104/pp.112.199729 23037507 PMC3510122

[21] Pietruszka M, Haduch-Sendecka A. Pressure-induced wall thickness variations in multi-layered wall of a pollen tube and Fourier decomposition of growth oscillations. Gen Physiol Biophys 2015; 34: 145–156. 10.4149/gpb_2014035 25675387

[22] Shipway and Shipway Solenoid properties. Inductance and magnetic B-field. 2008. http://www.calctool.org/CALC/phys/electromagnetism/solenoid (Date access: 15/05/2014).

