# Correction: Ion Frequency Landscape in Growing Plants

**DOI:** 10.1371/journal.pone.0143787

**Published:** 2015-11-23

**Authors:** Mariusz Pietruszka, Aleksandra Haduch-Sendecka

The incorrect reference is given in the S4 Fig caption. Please view the figure with the correct caption below.

References 22–35 are missing. Please view these references below.

22. Messerli MA, Danuser G, Robinson KR. Pulsative influxes of H^+^, K^+^ and Ca^2+^ lag growth pulses of *Lilium longiflorum* pollen tubes. J Cell Sci 1999; 112: 1497–1509.

23. Messerli MA, Creton R, Jaffe LF, Robinson KR. Periodic increases in elongation rate precede increases in cytosolic Ca2+ during pollen tube growth. Dev Biol 2000; 222: 84–98.

24. Holdaway-Clarke TL, Feijó JA, Hackett GR, Kunkel JG, Hepler PK. Pollen tube growth and the intracellular cytosolic calcium gradient oscillate in phase while extracellular calcium influx is delayed. Plant Cell 1997; 9: 1999–2010.

25. Cardenas L, McKenna ST, Kunkel JG, Hepler PK. NAD(P)H oscillates in pollen tubes and is correlated with tip growth. Plant Physiol 2006; 142: 1460–1468.

26. Cardenas L, Lovy-Wheeler A, Kunkel JG, Hepler PK. Pollen tube growth oscillations and intercellular calcium levels are reversibly modulated by actin polymerization. Plant Physiol 2008; 146: 1611–1621.

27. Kroeger JH, Daher FB, Grant M, Geitmann A. Microfilament orientation constrains vesicle flow and spatial distribution in growing pollen tubes. Biophys J 2009; 97: 1822–1831.

28. Michard E, Alves F, Feijo JA. The role of ion flues in polarized cell growth and morphogenesis: the pollen tube as an experimental paradigm. Int J Dev Biol 2009; 53: 1609–1622.

29. Liu J. Piette BMAG, Deeks MJ, Franklin-Tong VE, Hussey PJ. A compartmental model analysis of integrative and self-regulatory ion dynamics in pollen tube growth. PLoS ONE 2010; 5: e13157.

30. Chavarria-Krauser A,Yejie D. A model of plasma membrane flow and cytosis regulation in growing pollen tubes. J Theor Biol 2011; 285: 10–24.

31. Kroeger JH, Zerzour R, Geitmann A. Regulator or driving force? The role of turgor pressure in oscillatory plant cell growth. PLoS ONE 2011; 6: e1854. doi:10.1371/journal.pone.0018549.

32. Kochian LV, Shaff JE, Kühtreiber WM, Jaffe LF, Lucas WJ. Use of an extracellular, ion-selective, vibrating microelectrode system for the quantification of K+, H+, and Ca2+ fluxes in maize roots and maize suspension cells. Planta 1992; 12: 601–610.

33. Shabala SN, Newman IA, Morris J. Oscillations in H+ and Ca2+ ion fluxes around the elongation region of corn roots and effects of external pH. Plant Physiol 1997; 113: 111–118.

34. Shabala S, Newman I, Whittington J, Juswono U. Protoplast ion fluxes: their measurement and variation with time, position and osmoticum. Planta 1998; 204: 146–152.

35. Shipway A, Shipway S. Solenoid properties. Inductance and magnetic B-field. 2008. http://www.calctool.org/CALC/phys/electromagnetism/solenoid (Date access: 15/05/2014).

## Supporting Information

S4 Fig The solenoid that was used for measurements. The elongating zone of the coleoptile is shown in green. The figure is based on the Shipway and Shipway [35] solenoid properties calculator.
(TIF)
