# Supplementary figures and images for: Ion Frequency Landscape in Growing Plants
Source: PLoS One. 2015 Oct 7;10(10):e0138839. doi: 10.1371/journal.pone.0138839 (PMC4596807; doi:10.1371/journal.pone.0138839)

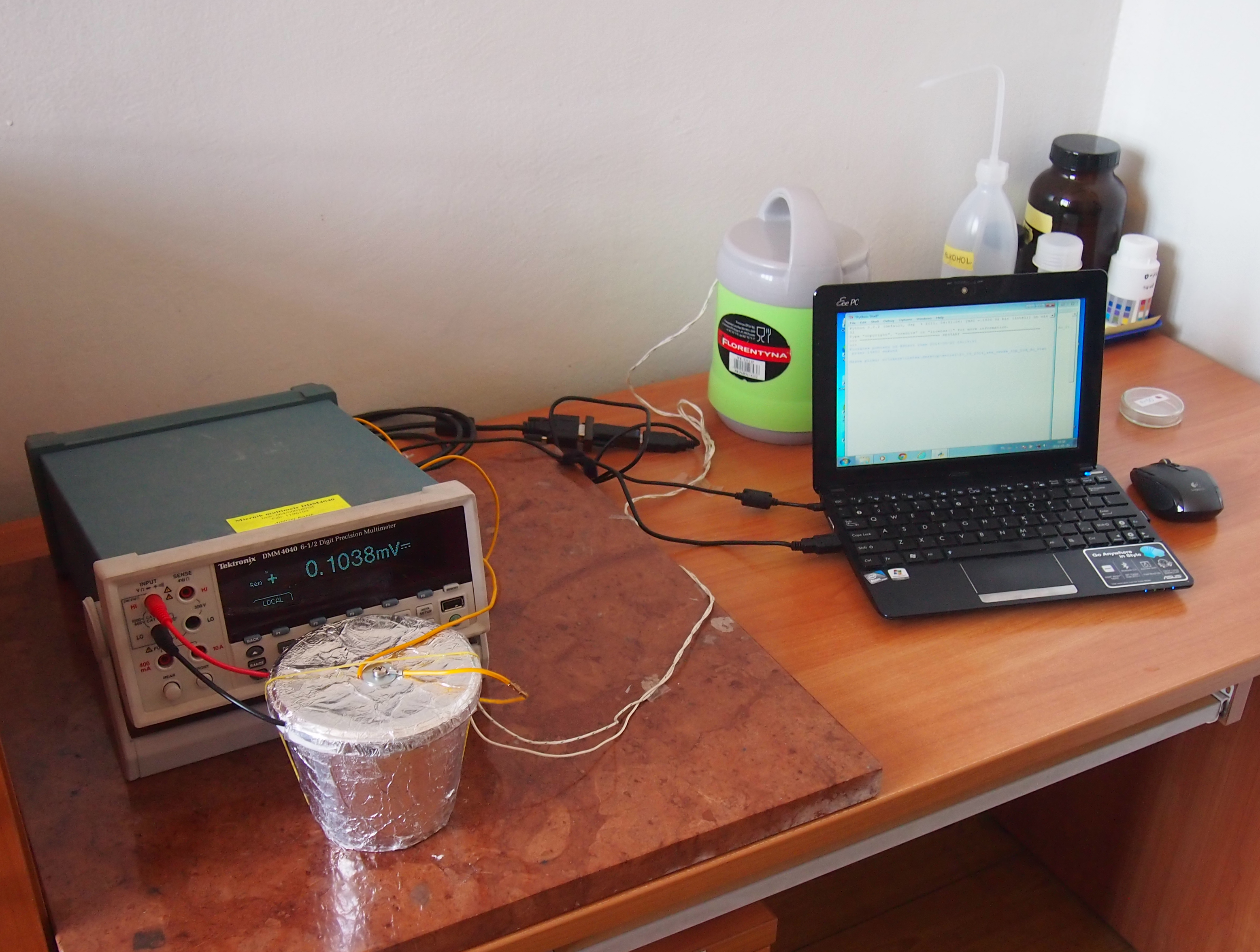

Supplement: S1 Fig — Growing maize (Zea mays L.) coleoptile was placed in a thermally isolated (Styrofoam) Faraday cage (Al film) that was situated on a heavy marble plate in order to filter high-frequency ground oscillations. (TIF) [file pone.0138839.s001.tif]

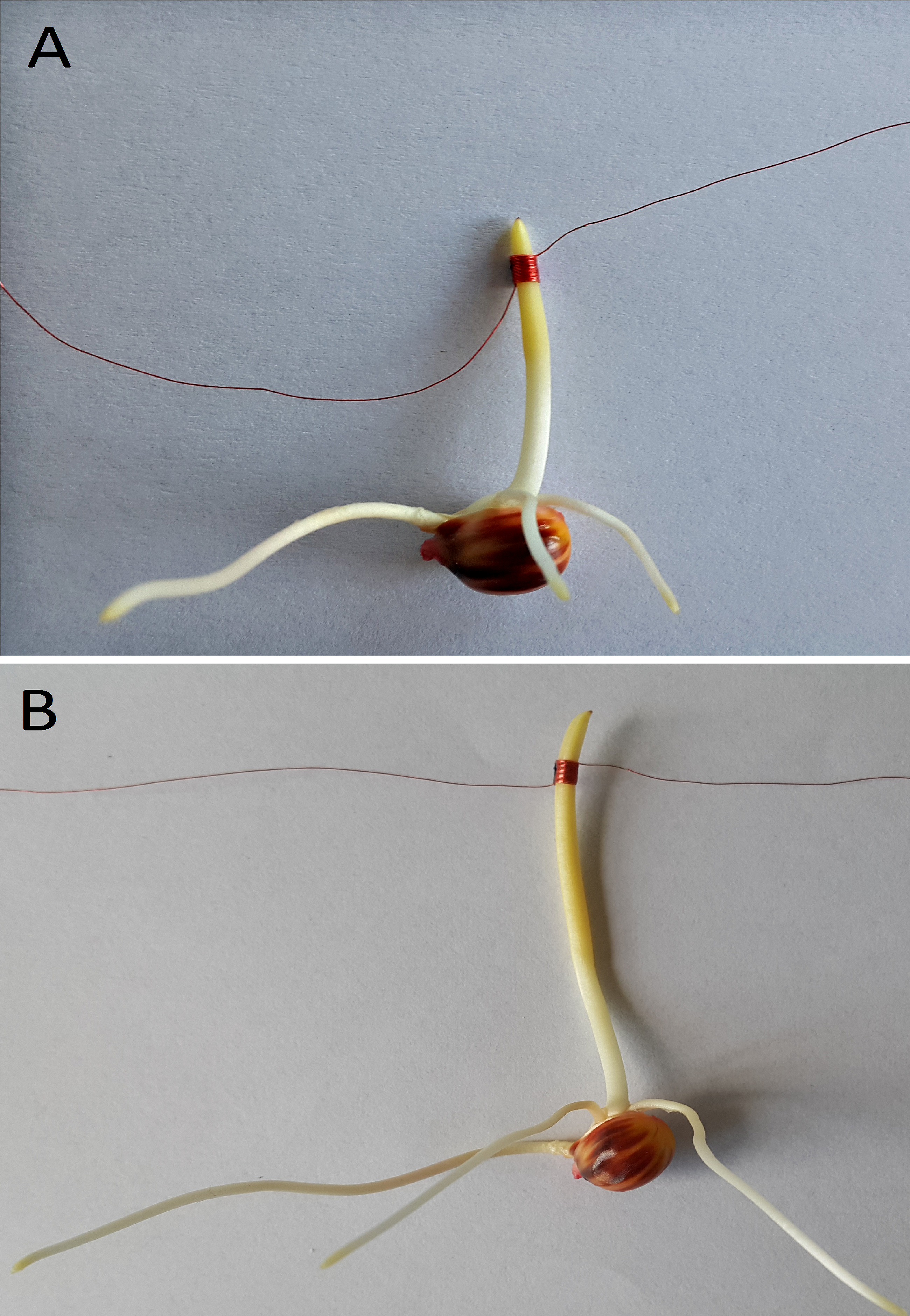

Supplement: S2 Fig — During the experiment the solenoid was moved from the tip to a more basal position of a coleoptile. The Cu connectors (wires), which were connected to the experimental setup, are visible. A. The investigated sample that was prepared for the experiment–the solenoid is initially placed 5 mm below the tip of a three-day-old seedling. B. Photograph taken after a 24 hours measurement. The solenoid was moved to a position of 6.25 mm below the tip. The coleoptile elongation equalled 14.85 mm during the experiment. (TIF) [file pone.0138839.s002.tif]

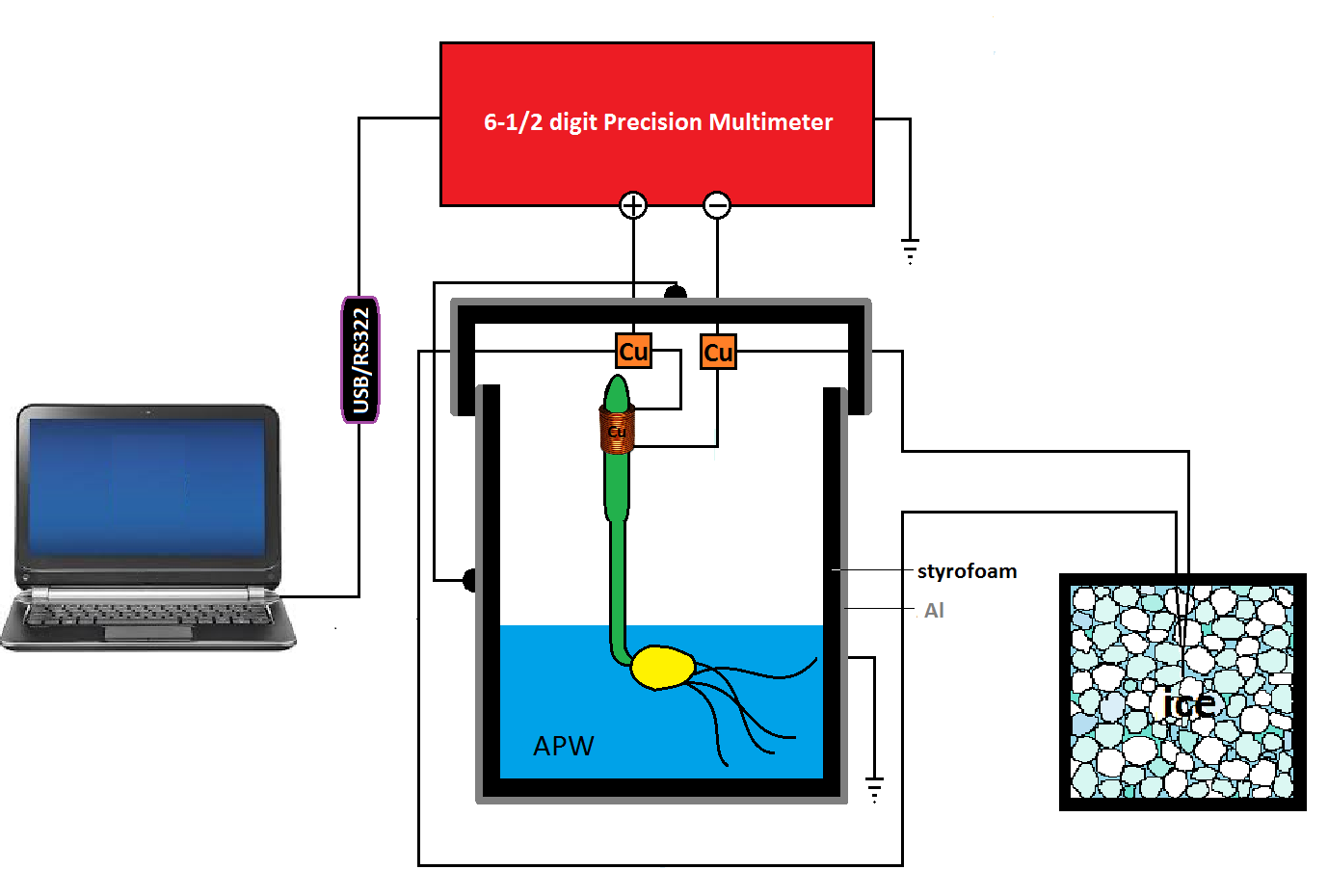

Supplement: S3 Fig — The voltage was measured and recorded through a RS232/USB interface and the data acquisition was completed on a computer using the Python code (.py). (PNG) [file pone.0138839.s003.png]

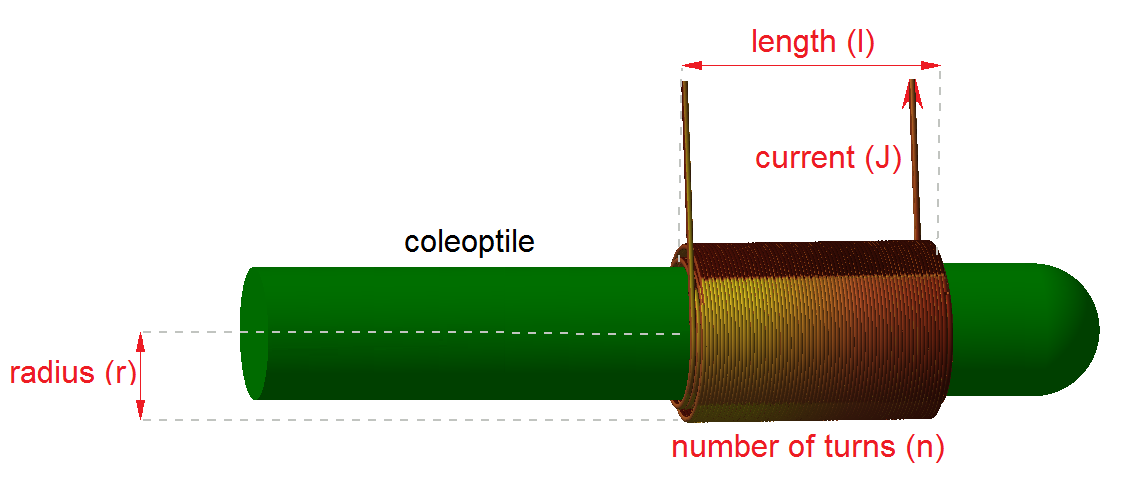

Supplement: S4 Fig — The elongating zone of the coleoptile is shown in green. The figure is based on the Shipway and Shipway [22] solenoid properties calculator. (TIF) [file pone.0138839.s004.tif]
