# Supplementary figures and images for: Correction: Ion Frequency Landscape in Growing Plants
Source: PLoS One. 2015 Nov 23;10(11):e0143787. doi: 10.1371/journal.pone.0143787 (PMC4658177; doi:10.1371/journal.pone.0143787)

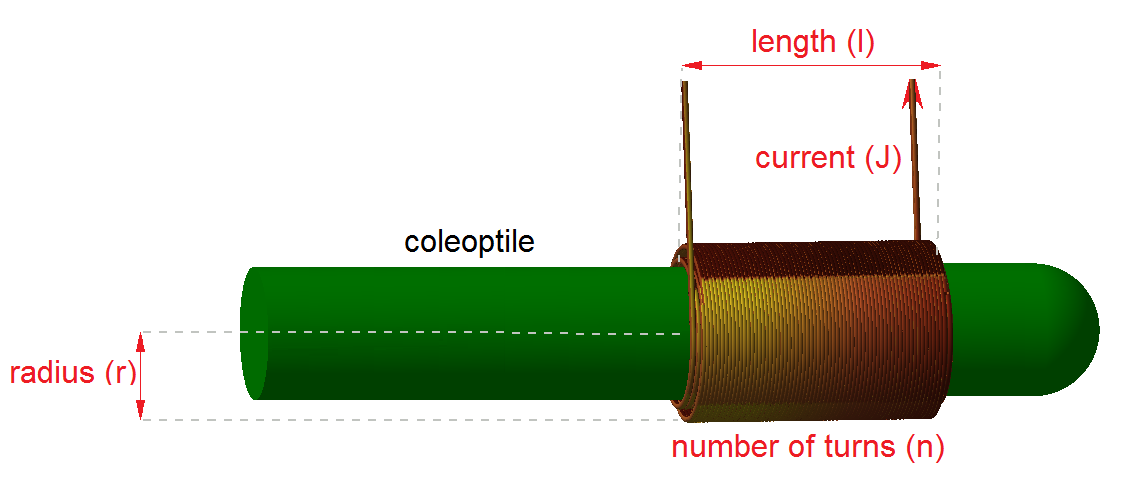

Supplement: S4 Fig — The elongating zone of the coleoptile is shown in green. The figure is based on the Shipway and Shipway [35] solenoid properties calculator. (TIF) [file pone.0143787.s001.tif]
